# Cas9 Contributes to Group B Streptococcal Colonization and Disease

**DOI:** 10.3389/fmicb.2019.01930

**Published:** 2019-08-21

**Authors:** Brady L. Spencer, Liwen Deng, Kathryn A. Patras, Zachary M. Burcham, Glenda F. Sanches, Prescilla E. Nagao, Kelly S. Doran

**Affiliations:** ^1^Department of Immunology & Microbiology, University of Colorado Anschutz Medical Campus, Aurora, CO, United States; ^2^Department of Biology, San Diego State University, San Diego, CA, United States; ^3^Department of Animal Sciences, Colorado State University, Fort Collins, CO, United States; ^4^Roberto Alcântara Gomes Biology Institute, Rio de Janeiro State University, Rio de Janeiro, Brazil

**Keywords:** *Streptococcus agalactiae*, Group B *Streptococcus*, meningitis, colonization, two-component systems, CRISPR/Cas, pathogenesis

## Abstract

Group B *Streptococcus* (GBS) is a major opportunistic pathogen in certain adult populations, including pregnant women, and remains a leading etiologic agent of newborn disease. During pregnancy, GBS asymptomatically colonizes the vaginal tract of 20–30% of healthy women, but can be transmitted to the neonate *in utero* or during birth resulting in neonatal pneumonia, sepsis, meningitis, and subsequently 10–15% mortality regardless of antibiotic treatment. While various GBS virulence factors have been implicated in vaginal colonization and invasive disease, the regulation of many of these factors remains unclear. Recently, CRISPR-associated protein-9 (Cas9), an endonuclease known for its role in CRISPR/Cas immunity, has also been observed to modulate virulence in a number of bacterial pathogens. However, the role of Cas9 in GBS colonization and disease pathogenesis has not been well-studied. We performed allelic replacement of *cas9* in GBS human clinical isolates of the hypervirulent sequence-type 17 strain lineage to generate isogenic Δ*cas9* mutants. Compared to parental strains, Δ*cas9* mutants were attenuated in murine models of hematogenous meningitis and vaginal colonization and exhibited significantly decreased invasion of human brain endothelium and adherence to vaginal epithelium. To determine if Cas9 alters transcription in GBS, we performed RNA-Seq analysis and found that 353 genes (>17% of the GBS genome) were differentially expressed between the parental WT and Δ*cas9* mutant strain. Significantly dysregulated genes included those encoding predicted virulence factors, metabolic factors, two-component systems (TCS), and factors important for cell wall formation. These findings were confirmed by qRT-PCR and suggest that Cas9 may regulate a significant portion of the GBS genome. We studied one of the TCS regulators, CiaR, that was significantly downregulated in the Δ*cas9* mutant strain. RNA-Seq analysis of the WT and Δ*ciaR* strains demonstrated that almost all CiaR-regulated genes were also significantly regulated by Cas9, suggesting that Cas9 may modulate GBS gene expression through other regulators. Further we show that CiaR contributes to GBS vaginal colonization and persistence. Altogether, these data highlight the potential complexity and importance of the non-canonical function of Cas9 in GBS colonization and disease.

## Introduction

*Streptococcus agalactiae* also known as Group B *Streptococcus* (GBS), is an important Gram-positive, β-hemolytic bacterial pathogen and a leading etiologic agent of neonatal invasive disease. GBS colonizes the gastrointestinal and urogenital tract of an estimated 20–30% of healthy individuals, including the vaginal tract of pregnant women (Wilkinson, 1978; Regan et al., 1991), and can be transmitted from carrier mothers to the newborn during birth (Schuchat, 1998). Up to 50–70% of vaginally-delivered babies of colonized mothers will become colonized and 1–2% of those colonized babies will develop invasive diseases, such as sepsis and meningitis (Fry, 1938; Schuchat, 1999; Campbell et al., 2000; Nandyal, 2008). Significant disease is caused by GBS, despite implementation of intrapartum antibiotic prophylaxis for colonized pregnant mothers (Leib and Tauber, 1999; Schrag et al., 2002; Chohan et al., 2006; Grandgirard and Leib, 2010), with 10–15% of invasive cases resulting in neonatal mortality (Gaschignard et al., 2011) and up to 40% of survivors developing permanent neurological sequelae including blindness, deafness, cerebral palsy, cognitive deficits, cerebral vascular assaults, and seizure activity (Edwards et al., 1985; Baraff et al., 1993; Arditi et al., 1998; Grimwood et al., 2000; Scheld et al., 2002). In particular, strains belonging to the lineage sequence type 17 (ST-17) have been implicated in severe GBS disease as they express an especially potent arsenal of virulence factors, such as the serotype III capsule and HvgA and serine-rich repeat protein-2 (Srr-2) adhesins. For this reason, ST-17 strains have been significantly associated with invasive neonatal disease (Poyart et al., 2008; Manning et al., 2009) and are becoming increasingly prevalent among immunocompromised adult populations, including the elderly and patients with diabetes or cancer (Pimentel et al., 2016; van Kassel et al., 2019).

As an opportunistic pathogen, GBS expresses a variety of surface and secreted factors in order to survive niche-specific stresses during vaginal colonization versus invasive disease. To colonize the vaginal tract, GBS attaches to the vaginal epithelium with surface-expressed adhesins such as Srr proteins and pili (Sheen et al., 2011; Wang et al., 2014), competes/co-exists with other vaginal normal flora (Chaisilwattana and Monif, 1995; Carson et al., 1997; Ortiz et al., 2014), and evades the host immune response (Kline et al., 2011; Patras et al., 2013). Under some circumstances, GBS can also ascend from the vagina to higher tissues such as the cervix and uterus and these infections are associated with adverse pregnancy outcomes (Seale et al., 2017a, b). Upon transmission of GBS to the neonate, however, other GBS factors may contribute to invasive disease progression. During the pathogenesis of meningitis, GBS expresses factors that promote survival in the bloodstream such as capsular polysaccharide (Hooven et al., 2018), the beta-hemolysin/cytolysin (β-h/c) and associated carotenoid pigment (Liu et al., 2004), as well as factors that promote interaction with the blood-brain barrier (BBB), such as surface adhesins BspC (Deng et al., 2019), SfbA (Mu et al., 2014) and pili (Maisey et al., 2007; Banerjee et al., 2011).

To adapt to new host environments, GBS requires regulatory mechanisms by which to modulate gene expression quickly, allowing production of essential bacterial factors. Two-component systems (TCS) are major sources of bacterial regulation and are comprised of a sensor histidine kinase, which senses cues from the environment, and a response regulator, which modulates gene expression (Hoch, 2000). In GBS, 21 TCS have been identified thus far (Glaser et al., 2002; Faralla et al., 2014), several of which have been studied, including RgfAC (Al Safadi et al., 2011), CiaRH (Quach et al., 2009; Mu et al., 2016), LiaR (Klinzing et al., 2013), CovRS (Jiang et al., 2005; Park et al., 2012), LtdR (Deng et al., 2018), SaeRS (Cook et al., 2018), and FspSR (Faralla et al., 2014). These TCS are thought to be important for expression of select bacterial factors in a niche-dependent manner. For example, the well-characterized TCS CovRS represses virulence factors such as β-h/c in acidic conditions (Park et al., 2012); therefore, it has been hypothesized to be an active repressor in acidic host niches, such as the vaginal tract (Santi et al., 2009). Additionally, SaeRS is up-regulated *in vivo* in GBS isolated from the murine vaginal tract compared to *in vitro* growth (Cook et al., 2018) and FspSR was found to mediate GBS vaginal persistence *in vivo* (Faralla et al., 2014). However, while many such TCS and their regulated genes have been shown to contribute to either colonization or pathogenesis (Park et al., 2012; Landwehr-Kenzel and Henneke, 2014; Doran et al., 2016; Deng et al., 2018), it is unclear how transcriptional regulators are coordinated to modulate global GBS gene expression in the varying niches of GBS colonization and disease progression (gastrointestinal tract, vaginal tract, placenta, lungs, blood, and brain). Therefore, the comprehensive mechanisms by which TCS and niche-specific virulence factors are regulated and how they promote GBS survival in various host environments warrant further study (Rajagopal, 2009).

Bacterial CRISPR (Clustered Regularly Interspaced Short Palindromic Repeats)/Cas systems provide defense against invading foreign nucleic acid (Marraffini, 2015). In Type II CRISPR interference, CRISPR-associated protein 9 (Cas9), an RNA-guided DNA endonuclease, complexes with a *trans-*activating small RNA (tracrRNA) and small guide CRISPR RNAs (crRNA, derived from the CRISPR array), and this ribonucleoprotein complex targets foreign invading DNA for degradation (Marraffini and Sontheimer, 2010). Recently, Cas9 has been associated with bacterial virulence, immune evasion, and interaction of pathogens with host cells via endogenous regulation of gene expression (Sampson and Weiss, 2013a, b; Dugar et al., 2018; Ma et al., 2018). However, the Cas9 regulon and the mechanism by which Cas9 regulates gene expression has only begun to be elucidated and varies between bacteria; therefore, further study into the conservation of the Cas9 regulon and regulatory mechanism across other *cas9-*encoding pathogens, such as GBS, is needed. A previous study on a GBS strain isolated from tilapia indicated that Cas9 influenced virulence (Ma et al., 2018); however, the role of Cas9 in both GBS colonization and disease in clinically relevant human isolates, such as those of ST-17 lineage, has not been determined.

In the present study, we sought to determine the role of Cas9 in the pathogenesis of GBS colonization and disease. We constructed Δ*cas9* mutants in clinical strains of the hypervirulent ST-17/serotype III lineage that were isolated from patients with invasive GBS disease and found that Δ*cas9* mutants were attenuated in murine models of hematogenous meningitis and vaginal colonization and exhibited a reduced ability to interact directly with host cells. We further observed that loss of Cas9 resulted in significant dysregulation of GBS genes including those encoding TCS, suggesting that Cas9 may modulate gene expression through other regulators.

## Results

### Construction of Δ*cas9* Mutants in ST-17 GBS Strains

**FIGURE 1:**
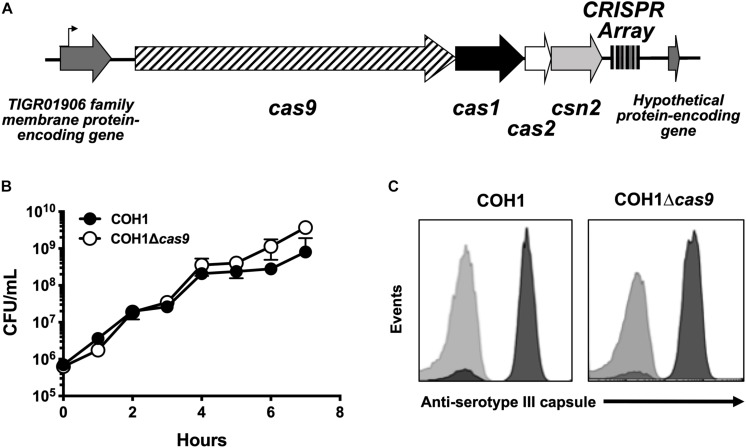
Characterization of a Δ*cas9* mutant in GBS. **(A)** Schematic of the GBS CRISPR/Cas region with varying number of CRISPR repeats within the CRISPR array. **(B)** The Δ*cas9* mutant grows normally in rich media and **(C)** expresses serotype III capsule, equivalent to wildtype COH1.

GBS contains a type II CRISPR/Cas locus, which includes the endonuclease effector Cas9 and additional CRISPR-associated genes: *cas1, cas2*, and *csn2* (involved in spacer acquisition during CRISPR interference) (Figure 1A; Nunez et al., 2014). Based on recent studies (Sampson et al., 2013; Dugar et al., 2018; Shabbir et al., 2018), we hypothesized that this system would contribute to GBS pathogenesis. To investigate the role of Cas9 specifically, we generated Δ*cas9* mutants in ST-17 strains by precise allelic-exchange mutagenesis as described previously (Jeng et al., 2003) and in the section “Materials and Methods.” One Δ*cas9* mutant was created in the strain COH1, a widely used GBS clinical isolate derived from a case of neonatal invasive disease (Kuypers et al., 1989). The deletion of *cas9* did not affect GBS growth in rich media or capsule production (Figures 1B,C). A second Δ*cas9* mutant was made in ST-17 strain GBS1428, which was isolated in 2014 from the urine of a 71-year-old male rectal cancer patient at Instituto Nacional do Cancer (INCA) in Rio de Janeiro, Brazil (Sanches and Nagao, unpublished). The GBS1428Δ*cas9* mutant exhibited similar growth kinetics in rich media compared to the WT parental strain (data not shown).

### Cas9 Contributes to Both GBS Disease Progression and Vaginal Persistence

**FIGURE 2:**
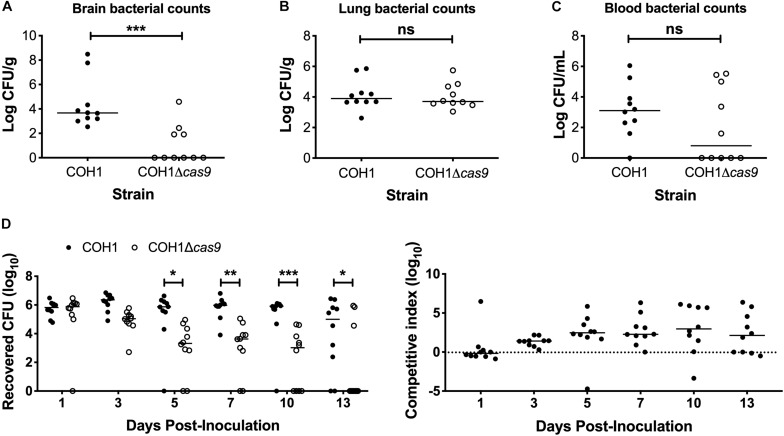
Cas9 mediates GBS disease progression and vaginal colonization, *in vivo*. Recovered bacterial CFU (WT COH1 or COH1Δ*cas9* mutant) in the **(A)** brain, **(B)** lungs, and **(C)** blood of CD-1 outbred mice at 72 h post-intravenous infection. Mann–Whitney *U* test *^∗∗∗^p* < 0.001. **(D)** Bacterial load of COH1 and COH1Δ*cas9* mutant in the vaginal tracts of C57BL/6 mice (*n* = 10/group) over time. Two-way repeated measures ANOVA with Sidak’s multiple comparisons, ^∗^*p* < 0.05; ^∗∗^*p* < 0.01; *^∗∗∗^p* < 0.001.

To determine if Cas9 plays a role in GBS virulence and disease progression, we utilized a model of GBS meningitis (Doran et al., 2003; Quach et al., 2009; Banerjee et al., 2011; Kim et al., 2015) that mimics hematogenous dissemination, resulting in pathogen brain penetration (Doran et al., 2003, 2005; van Sorge et al., 2008). We observed that the COH1Δ*cas9* mutant exhibited a decreased ability to penetrate brain tissue compared to WT COH1, while no significant differences were observed in recovered CFU from lung tissue (Figures 2A,B). Slight differences in recovered CFU from blood were observed between mice infected with WT COH1 and COH1Δ*cas9*, although these differences were not significant (Figure 2C). We also investigated the role of Cas9 in GBS colonization of the vaginal tract using our well-characterized murine model of GBS vaginal carriage (Sheen et al., 2011; Cavaco et al., 2013; Patras et al., 2013, 2015a,b; Patras and Doran, 2016). We observed in competition experiments that while both WT and mutant strains were able to initially colonize the vaginal tracts of C57BL/6 mice, the WT COH1 strain was able to out-compete the COH1Δ*cas9* mutant and therefore persisted longer and at higher bacterial loads than the COH1Δ*cas9* mutant (Figure 2D). Similar results were observed when using a different mouse (CD-1) background (Supplementary Figure S1A). The GBS1428Δ*cas9* mutant also exhibited decreased vaginal persistence and brain tissue penetration, with additionally decreased bacterial load in lung tissues and blood compared to the WT GBS1428 strain (Supplementary Figures S1B,C). Taken together, these data indicate that Cas9 contributes to both GBS virulence and colonization. As the COH1 strain has been well characterized and fully sequenced (Glaser et al., 2002; Tettelin et al., 2005; Da Cunha et al., 2014), we continued further studies with this strain background.

### Cas9 Contributes to GBS Interaction With Host Cells

**FIGURE 3:**
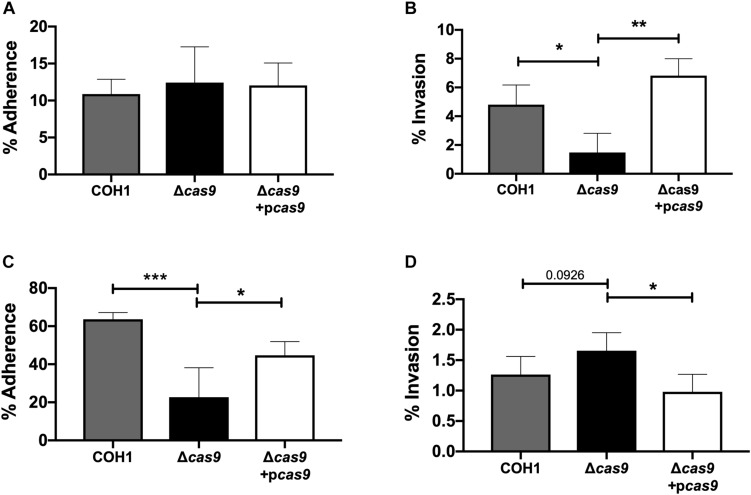
Cas9 mediates GBS interaction with host cells, *in vitro.* WT GBS, Δ*cas9* mutant, and Δ*cas9* + p*cas9* strains were used to assess **(A)** adherence to hCMEC, **(B)** invasion of hCMEC, **(C)** adherence to hVEC, and **(D)** invasion of hVEC. All data are represented as percent CFU recovered of the initial inoculum and were performed in technical replicates of *n* = 4. Each plot is representative of three individual experiments. One-way ANOVA with Sidak’s multiple comparisons, with comparisons made to the Δ*cas9* mutant, ^∗^*p* < 0.05; ^∗∗^*p* < 0.01; *^∗∗∗^p* < 0.001.

Our results suggest a role for Cas9 in GBS invasion and penetration into the brain as well as persistence in the vaginal tract. We hypothesized that these *in vivo* phenotypes may be due to a reduced ability of GBS to interact with host cells. Thus, we examined the ability of parental WT, Δ*cas9* mutant, and complemented strains to adhere to and invade human brain endothelium and vaginal epithelium, *in vitro*. To model the human BBB we utilized immortalized human cerebral microvascular endothelial cells (hCMEC), which maintain the morphologic and functional characteristics of primary brain endothelium (Weksler et al., 2005) and have proven valuable in the analysis of many human CNS disease-causing pathogens, including GBS (Huang et al., 2008; Vu et al., 2009; Fletcher et al., 2012; Krishnan et al., 2015; Wang et al., 2015). Similarly, a human vaginal epithelial cell line (hVEC) has been utilized as a model of female genital tract colonization by human pathogens such as GBS (Kucknoor et al., 2005; Peterson et al., 2005; Hollmer et al., 2006; Patras et al., 2015a). Using our standard GBS adhesion and invasion assays (Deng et al., 2018), an inoculum of 10^5^ CFU/well (multiplicity of infection [MOI] of 1.0) was added to hCMEC or hVEC monolayers. For adherence assays, bacteria were incubated with host cells for 30 min. For invasion assays bacteria were incubated with host cells for 2 h followed by an additional 2 h incubation with antibiotic treatment to kill extracellular bacteria. In both assays, bacteria were recovered, enumerated by serial dilutions and CFU count, and expressed as percent recovered GBS relative to the original inoculum. These studies demonstrated that the Δ*cas9* mutant adhered well to hCMEC, but exhibited a significant reduction (∼3-fold) in invasion into brain endothelium compared to WT GBS (Figures 3A,B). Unlike what we observed in hCMEC, the Δ*cas9* mutant exhibited a marked reduction in adherence to hVEC cells, while invasion was similar to that of WT COH1 (Figures 3C,D). Both phenotypes were complemented by plasmid expression of *cas9* in the Δ*cas9* mutant strain. Collectively, these data indicate that the attenuated phenotypes of the Δ*cas9* mutant during *in vivo* models of GBS colonization and disease may be partially due to its decreased association with host cells compared to WT GBS.

### Impact of Cas9 on GBS Gene Expression

**FIGURE 4:**
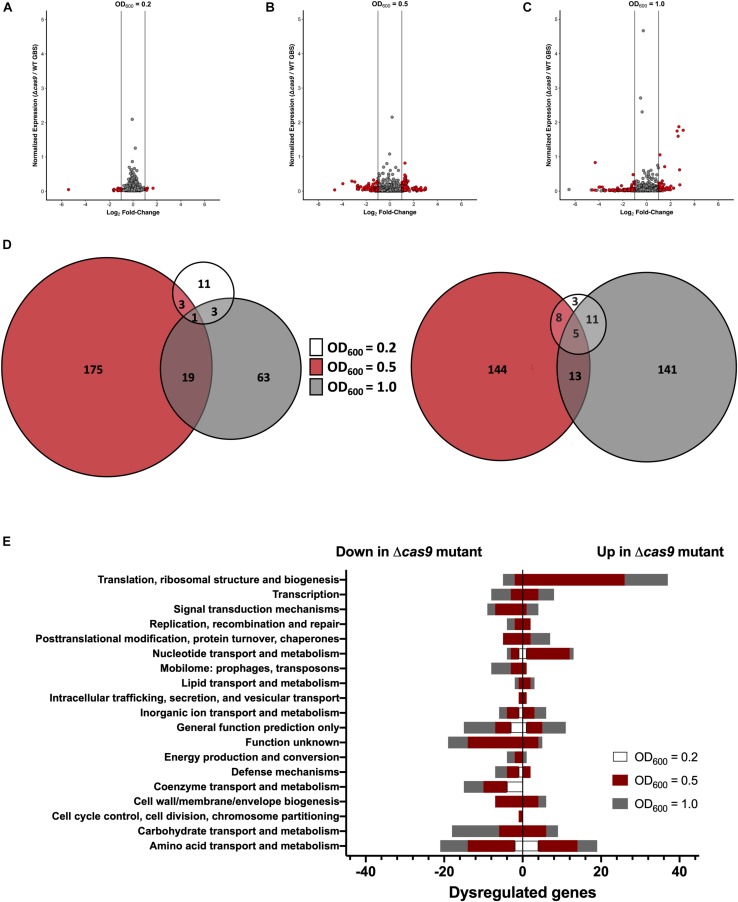
Cas9 globally regulates the GBS genome. RNA-Seq analysis was performed on RNA isolated from COH1 and COH1Δ*cas9* cultures grown in THB to early-, mid- and late-log growth phases (OD_600_ = 0.2, 0.5, and 1.0, respectively). Three biological replicates were analyzed for each strain. Volcano plots indicate combined analysis from DESeq2 and EdgeR at **(A)** OD_600_ = 0.2, **(B)** OD_600_ = 0.5, and **(C)** OD_600_ = 1.0. Red dots indicate hits that were significantly dysregulated (*p* < 0.05, fold change >±2). Gray dots indicate non-significant hits. **(D)** Total number of dysregulated genes between COH1 and COH1Δ*cas9* strains at each growth phase by Venn diagram. The Venn diagram on the left indicates the number of genes that are up-regulated in the Δ*cas9* mutant compared to WT COH1 and the Venn diagram on the right indicates the number of genes that are down-regulated in the Δ*cas9* mutant compared to WT COH1. **(E)** Clusters of Orthologous Groups of proteins designations assigned to significantly dysregulated genes in the Δ*cas9* mutant compared to WT COH1, as assigned by Integrated Microbial Genomes & Microbiomes (IMG/M) system at the JGI. Bars to the left indicate genes that are down-regulated in the Δ*cas9* mutant compared to WT COH1, whereas bars to the right indicate genes that are up-regulated in the Δ*cas9* mutant compared to WT COH1.

Previous studies have shown that Cas9 can mediate endogenous gene regulation (Sampson and Weiss, 2013b; Ma et al., 2018); thus, we hypothesized that Cas9 may affect factors important for GBS colonization and disease progression. To determine if Cas9 modulates GBS gene expression, we performed RNA-Seq analysis to compare the transcriptome of WT and Δ*cas9* mutant strains during various growth phases in rich media. GBS growth was monitored by measuring optical density and RNA was collected from cultures grown to OD_600_ of 0.2, 0.5, and 1.0, corresponding to early-, mid-, and late-log growth phases (Supplementary Figure S2). Library construction, sequencing, and analysis were performed at the Broad Institute as described in Methods and analysis was conducted using both DESeq2 and EdgeR (Love et al., 2014). Significant global changes (≥2-fold, *p* < 0.05) were observed in the Δ*cas9* mutant compared to WT COH1 at all growth phases (Figures 4A–C), with the highest number of significantly differentially expressed genes identified at exponential phase (OD_600_ = 0.5) (Figure 4B). Altogether, 353 of 2045 coding sequences (GenBank accession NZ_HG939456.1), or >17% of the GBS genome, were dysregulated in the COH1Δ*cas9* mutant compared to WT COH1. When separated by growth phase, the number of significantly perturbed genes was 22 at early log (OD_600_ = 0.2; Supplementary Table S1), 245 at mid-log (OD_600_ = 0.5; Supplementary Table S2), and 157 at late-log (OD_600_ = 1.0; Supplementary Table S3 and Figures 4A–D). These findings suggest that Cas9 may globally regulate the GBS genome. Dysregulated genes were grouped by function using the “Cluster of Orthologous Groups of proteins” (COGs) designations provided by Integrated Microbial Genomes & Microbiomes (IMG/M) system at the Joint Genome Institute (JGI) (Chen et al., 2019). Broadly, genes assigned functions in “translation and ribosome biogenesis” and “nucleotide metabolism and transport” were up-regulated in the COH1Δ*cas9* mutant compared to COH1, while genes assigned functions in “carbohydrate transport,” “coenzyme transfer,” and “signal transduction” were largely down-regulated in the COH1Δ*cas9* mutant compared to COH1 (Figure 4E).

**FIGURE 5:**
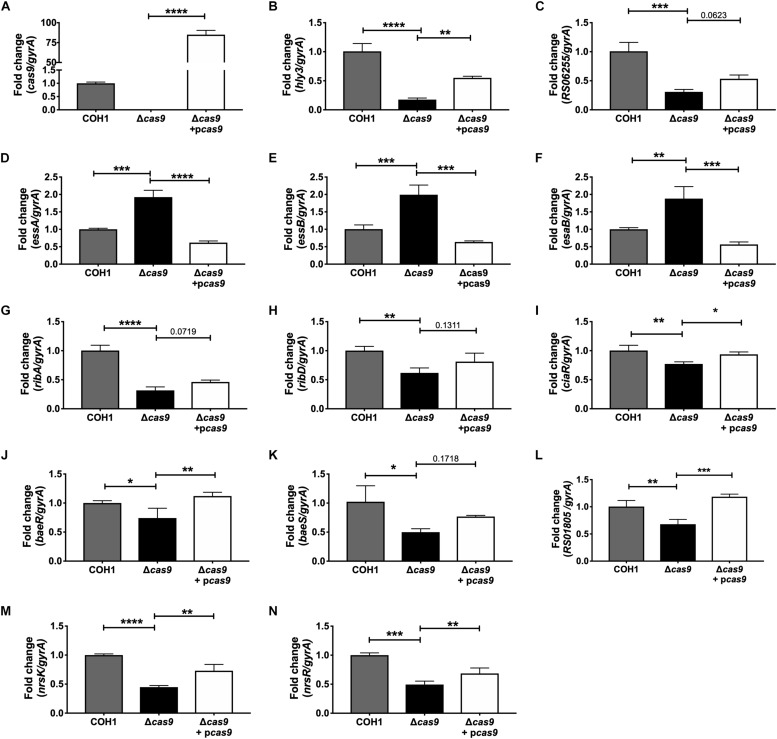
qRT-PCR confirmation of RNA-Seq analysis. Transcript abundances of significantly dysregulated genes identified in RNA-Seq analysis were confirmed in WT, Δ*cas9*, and Δ*cas9* + p*cas9* strains. **(A–N)** Fold change was calculated using the ΔΔCT equation. *n* = 3 cultures, One-way ANOVA with Sidak’s multiple comparisons, with comparisons made to the Δ*cas9* mutant, ^∗^*p* < 0.05; ^∗∗^*p* < 0.01; ^∗∗∗^*p* < 0.001; ^∗∗∗∗^*p* < 0.0001.

Within this data set, we identified many significantly dysregulated genes encoding GBS factors that have not been characterized in GBS. These included genes encoding predicted virulence factors (hemolysin III, type VII secretion machinery), metabolic factors (riboflavin biosynthesis, iron transport, sugar metabolism, ATP synthesis), TCS, and factors important for cell wall formation (Supplementary Tables S1–S3). Representative genes to confirm by qRT-PCR were chosen based on level of dysregulation and on novelty of function in GBS pathogenesis. Expression of *cas9* was first confirmed in COH1, COH1Δ*cas9* mutant, and complemented strains (Figure 5A). Other highly dysregulated genes were also confirmed by qRT-PCR, including genes encoding hemolysin III (*hly3*) and its upstream putative regulator (*RS06255*), genes encoding for type VII secretion components (*essA, essB, esaB*), and genes encoding riboflavin synthesis machinery (*ribA* and *ribD*) (Figures 5B–H). Finally, many transcriptional regulators were identified within the RNA-Seq dataset as well, including three TCS that were significantly down-regulated in the Δ*cas9* mutant compared to WT (Figures 5I–N). Two of the TCS have been previously characterized or described: CiaRH, which has been shown to promote GBS virulence and survival during intracellular stress (Quach et al., 2009) and NsrRK (Khosa et al., 2013), which promotes GBS nisin resistance. A third TCS identified in this data set that has been previously identified but not studied in GBS (Faralla et al., 2014) has homology to BaeSR in *Escherichia coli* (Leblanc et al., 2011; Wang and Fierke, 2013). Finally, gene *RS01805* (annotated as a sensor histidine kinase in GenBank accession NZ_HG939456.1) was down-regulated in the Δ*cas9* mutant compared to WT COH1. *RS01805* shares homology with *luxS* in *Streptococcus pyogenes* and LuxS has been shown to regulate expression of S. *pyogenes* factors involved in its internalization by epithelial cells. These representative dysregulated genes could be either fully or partially complemented with the *cas9* overexpression strain. We observed similar dysregulation of many of these genes in the GBS1428Δ*cas9* mutant compared to the parental GBS1428 strain (Supplementary Figure S3). Taken together, these results indicate that Cas9 deficiency results in global transcriptional changes, which may promote GBS vaginal colonization and BBB penetration.

### Impact of CiaR on GBS Transcriptome and Vaginal Persistence

**FIGURE 6:**
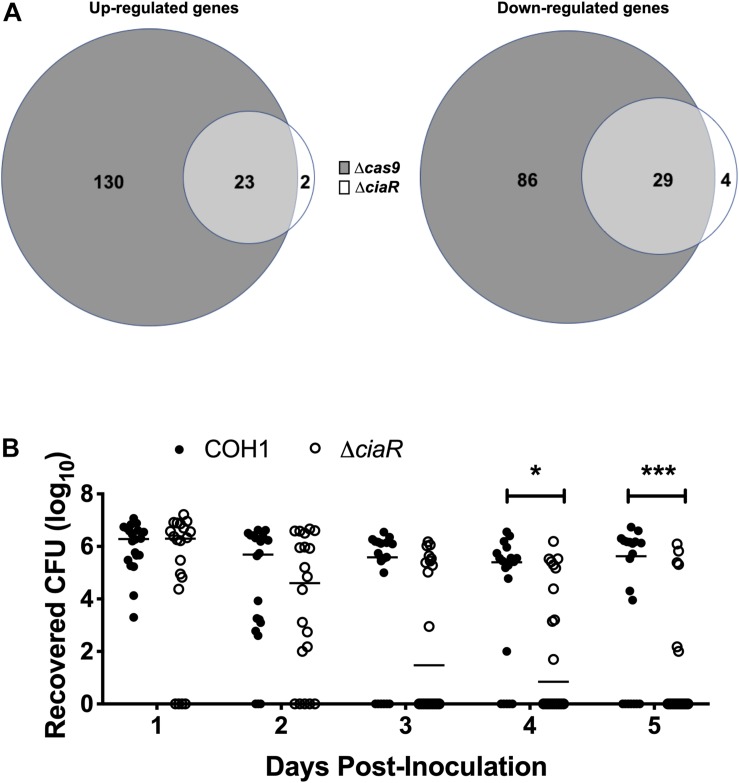
Cas9 regulates two component system response regulator CiaR, which contributes to GBS persistence within the vaginal tract. **(A)** Total number of dysregulated genes between COH1Δ*cas9* and COH1Δ*ciaR* strains at each mid-log phase by Venn diagram. Venn diagram on the left indicate number of genes that are up-regulated in the Δ*cas9* or Δ*ciaR* mutants compared to WT COH1 and Venn diagrams on the right indicate number of genes that are down-regulated in the Δ*cas9* or Δ*ciaR* mutants compared to WT COH1. Three biological replicates were analyzed for each strain. **(B)** Bacterial load of COH1 and COH1Δ*ciaR* mutant in the vaginal tracts of CD-1 outbred (*n* = 20/group) over time. Two-way repeated measures ANOVA with Sidak’s multiple comparisons, ^∗^*p* < 0.05; ^∗∗∗^*p* < 0.001.

As we observed a down-regulation of TCS in the Δ*cas9* mutant, we hypothesized that Cas9 may impact the GBS transcriptome through other regulators. CiaR has been studied previously and a few CiaR-regulated genes were identified previously by microarray analysis (Quach et al., 2009). To determine the full transcriptome of the Δ*ciaR* mutant compared to WT COH1, we analyzed strains grown to mid-log by RNA-Seq, as described above. We observed that 58 genes were significantly dysregulated in the Δ*ciaR* mutant compared to WT COH1. Of the 25 genes upregulated in the Δ*ciaR* mutant compared to WT COH1, 23 were also up-regulated in the Δ*cas9* mutant. Similarly, of the 33 genes down-regulated in the Δ*ciaR* mutant compared to WT COH1, 29 were also down-regulated in the Δ*cas9* mutant (Figure 6A and Supplementary Table S4). Genes that were significantly down-regulated in both the Δ*cas9* and Δ*ciaR* mutants compared to WT COH1 included those previously reported to be regulated by CiaR, *SAN_2180* and *SAN_0039* (annotated as *RS09565* and *RS00315* in our data sets, respectively) (Quach et al., 2009).

*SAN_0039* encodes for a putative bacteriocin-like inhibitory substance (BLIS), which shares 56% identity with a well-characterized BLIS encoded by *Streptococcus zooepidemicus* (Simmonds et al., 1996; Lai et al., 2002; Akesson et al., 2007). GBS has been shown to inhibit common vaginal bacteria such as streptococci, *Lactobacillus* spp., and *Gardnerella vaginalis, in vitro* (Chaisilwattana and Monif, 1995); therefore, we hypothesized that CiaR-regulation of GBS factors such as *SAN_0039* (and/or other CiaR-regulated factors) may affect the ability of GBS to compete with the vaginal normal flora and establish vaginal colonization. To determine if CiaR regulation might contribute to GBS persistence within the vaginal tract, we examined vaginal colonization with WT COH1 and Δ*ciaR* strains and observed that the Δ*ciaR* mutant was, in fact, cleared more rapidly from the vaginal tract compared to the WT strain (Figure 6B).

## Discussion

In this study, we have shown that Cas9 contributes to both GBS colonization of the vaginal tract as well as penetration into the brain during GBS disease progression. We also showed that a GBSΔ*cas9* mutant was attenuated in interaction with human cells, exhibiting decreased attachment to vaginal epithelium and reduced invasion of brain microvascular endothelial cells. This is consistent with previous studies showing that Cas9 contributes to host cell interaction by other meningeal pathogens, such as *Neisseria meningitidis* (Sampson et al., 2013) and a tilapia-derived strain of GBS (Ma et al., 2018). Since previous studies in other human bacterial pathogens have described a role for Cas9 in endogenous regulation of the bacterial genome, we investigated this putative function for Cas9 in clinical isolates of GBS. Using RNA-Seq analysis we determined that greater than 17% of the genome was dysregulated in a GBSΔ*cas9* mutant at the transcriptomic level. Given the phenotypes of the Δ*cas9* mutant in adherence to and invasion of brain endothelial and vaginal epithelial cells, we expected to observe dysregulation of genes encoding for cell wall anchored proteins or for known or predicted GBS adhesins in the Δ*cas9* mutant compared to WT COH1. However, very few genes in this category were found to be altered, similar to recently published transcriptomic analysis of *cas9*-deficient strains in *N. meningitidis* (Heidrich et al., 2019), in which Cas9 was shown to affect adherence to epithelial cells indirectly, not by modulating expression of adhesins.

Of special interest, many of the highly dysregulated genes in the Δ*cas9* mutant compared to WT were largely unstudied in GBS. These included predicted virulence factors, such as hemolysin III and factors involved in riboflavin synthesis and type VII secretion. Hemolysin III has been characterized in *Bacillus* and *Vibrio* species as a pore-forming hemolysin, specific for human red blood cells (Baida and Kuzmin, 1995; Chen et al., 2004). Two-component system response regulator LtdR was shown to modulate expression of the *hly3* gene previously (Deng et al., 2018), but hemolysin III has not been characterized specifically in the pathogenesis of GBS. Riboflavin synthesis has also not been examined in GBS, but has been shown to modulate iron acquisition and host response in other bacterial species (Worst et al., 1998; Crossley et al., 2007). Finally, our RNA-Seq analysis identified that genes encoded within the type VII secretion system operon, which has been characterized in *Staphylococcus aureus* (Unnikrishnan et al., 2017) but not GBS, were significantly dysregulated in the Δ*cas9* mutant. These factors will be of interest to examine in follow-up studies.

In recent years, the novel endogenous regulation function of Cas9 has been studied in *Francisella novicida*, *Campylobacter jejuni*, and in a tilapia-derived strain of *S. agalactiae* (Sampson and Weiss, 2013a, b; Dugar et al., 2018; Ma et al., 2018). In these studies, complementary binding regions of various small RNAs were identified that corresponded with endogenous nucleic acids. In *F. novicida* studies, Cas9-mediated regulation (at the DNA level) was lost upon disruption of these complementary regions, indicating that direct binding of complementary small RNAs (complexed with Cas9) to endogenous DNA can result in transcriptional repression (Ratner et al., 2019). In *C. jejuni*, Cas9 was shown to target and degrade endogenous mRNA, which was also dependent on complementarity of the CRISPR RNA to endogenous targets (Dugar et al., 2018). Regardless of the mechanism, however, in all studies of Cas9-mediated regulation to date, the Cas9 regulons described have been limited to just a few genes. For example, in *F. novicida* two transcripts were dysregulated in a Δ*cas9* mutant (Ratner et al., 2019) and in tilapia-derived GBS 29 genes were found to be dysregulated in the Δ*cas9* mutant, with 16 of those found within a prophage (Ma et al., 2018). Upon RNA-Seq analysis of clinically relevant COH1 and its isogenic Δ*cas9* mutant, we found that 353 genes were significantly dysregulated. Our study is the first to show that Cas9 deficiency results in global transcriptional changes in a clinically-relevant human GBS isolate. COH1 and GBS1428 strains contain five and six CRISPR spacers, respectively, five of which are shared between the two strains (Grissa et al., 2007), Supplementary Table S6). However, we could not find significant complementary sequences uniquely between the CRISPR RNAs/repeats and GBS genomic regions that were dysregulated in the Δ*cas9* mutants, indicating that the GBS Cas9 may function differently than has been described in other bacteria. These data suggest that there may be multiple mechanisms (CRISPR array dependent and independent) by which Cas9 regulates bacterial gene expression, whether at the genomic DNA or mRNA level. While we have not assessed the abundance of small RNAs or their complementarity to endogenous GBS DNA in the present study, this analysis would be useful for future investigation into the mechanism of Cas9-mediated regulation in GBS.

Because a large portion of the genome was dysregulated in our Cas9-deficient strain, we speculate that it is unlikely that Cas9 interacts with the promoter regions of all the affected genes directly. While this remains to be demonstrated experimentally, we propose here that Cas9 may modulate expression of TCS and other regulators, facilitating GBS adaptation to diverse host niches. GBS regulatory factors have been well described (Rajagopal, 2009) and it is well-appreciated that GBS gene expression is extremely fine-tuned given the 21 TCS found in GBS thus far (Faralla et al., 2014). At mid-log, three TCS were found to be significantly downregulated in the Δ*cas9* mutant compared to COH1, namely CiaRH, NsrRK, and a TCS with homology to BaeSR, which has been characterized in *E. coli* (Figures 5J–L and Supplementary Table S2). We have assessed this gene regulation role for Cas9 in clinically relevant ST-17 GBS strains, but it is important to note that the Cas9 regulon may be largely strain-dependent, as has been shown for another global regulator in GBS, CovRS (Jiang et al., 2008). Over 100 genes were dysregulated by CovR in GBS strain NEM316 (Lamy et al., 2004), however, following transcriptomic analysis in two other clinical isolate strains, just 39 genes were determined to comprise the CovRS core regulon (Jiang et al., 2008). Indeed, the Cas9 regulon for the tilapia-derived GBS strain previously described consisted of just 29 genes, with 16 of those found within a prophage (Ma et al., 2018). This is unsurprising as the genomes of fish-derived GBS have been shown in multiple studies to drastically differ in content from genomes of human isolates, especially in TCS (Rosinski-Chupin et al., 2013; Faralla et al., 2014). Since we propose here that Cas9 modulation of TCS may drive global gene expression differences, a large variation in TCS between fish and human strains would almost certainly impact the Cas9 regulon.

The GBS response regulator CiaR has been shown to modulate GBS virulence and interaction with host cells (Quach et al., 2009; Mu et al., 2016). Importance for CiaRH in virulence has also been well-described in pneumococcus and oral streptococci (Qi et al., 2004; Blanchette-Cain et al., 2013; Trihn et al., 2013; Davey et al., 2016; Hentrich et al., 2016; Zhu et al., 2017). CiaRH expression is down-regulated in GBSΔ*cas9* mutants (Figure 5I, Supplementary Figure S2, and Supplementary Table S2); therefore, we hypothesized that CiaR-regulated genes would also be modulated in the Δ*cas9* mutant compared to WT COH1. We performed RNA-Seq analysis of a Δ*ciaR* mutant compared to WT COH1 and found that 53 of the 58 significantly dysregulated genes in the Δ*ciaR* mutant were also dysregulated in the Δ*cas9* mutant. This indicates that Cas9 regulation may result in a cascade effect, causing the dysregulation of many regulator’s regulons. In addition to CiaRH, other TCS were regulated by Cas9, which would be of interest for future studies. A TCS unstudied in GBS (homologous to BaeSR) was identified in *E. coli* and shown to be important for antibiotic resistance, limiting zinc toxicity, and prevention of envelope stress (Leblanc et al., 2011; Wang and Fierke, 2013), and the NsrRK TCS was shown to confer nisin resistance in GBS (Khosa et al., 2013, 2016). Dysregulation of these TCS regulators in our GBSΔ*cas9* mutants may therefore promote the observed attenuation of the Δ*cas9* mutants *in vivo*. Future studies aim to elucidate the exact mechanism of Cas9 regulation as well as characterize the contribution of specific factors in GBS pathogenesis. This will ultimately provide mechanistic insights into the non-canonical roles of Cas9 during pathogen colonization and disease, with the potential to identify new targets for therapeutic intervention.

## Materials and Methods

### Bacterial Strains and Cell Lines

*Escherichia coli* strain MC1061 (Mclab) was used to propagate the empty pDCErm plasmid and the pDCErm:*cas9* overexpression plasmid, pBLS2001. pDCErm-containing *E. coli* strains were grown in LB (Luria broth; Research Products International, RPI) supplemented with 500 μg/mL erythromycin at 37°C, shaking. All GBS strains were derived from ST-17/serotype III clinical isolates COH1 (Kuypers et al., 1989) or GBS1428 (Sanches and Nagao, unpublished). GBS strains were grown in Todd Hewitt Broth (THB; Research Products International, RPI), statically at 37°C. When necessary, GBS strains were grown in THB + 2 μg/mL chloramphenicol or THB + 5 μg/mL erythromycin. The human microvascular endothelial cell line utilized in this study was purchased from Millipore and grown in EndoGRO-MV complete medium kit (catalog # SCME004) (Weksler et al., 2005; Vu et al., 2009) supplemented with 1 ng/mL Fibroblast growth factor-2 (FGF-2). The hVEC used in this study was purchased from American Type Culture Collection (VK2/E6E7, ATCC CRL-2616) and grown in Keratinocyte serum-free medium (KSFM; Gibco) supplemented with 0.1 ng/ml human recombinant epidermal growth factor (EGF; Gibco) and 0.05 mg/ml bovine pituitary extract (BPE; Gibco). Both cell lines were grown at 37°C with 5% CO_2_.

### Construction of GBS Mutants and Complemented Strains

In-frame deletion mutants of *cas9* were made by allelic exchange as previously described (Jeng et al., 2003). Briefly, 5′ and 3′ flanking regions surrounding *cas9* (*RS04480* in GenBank accession NZ_HG939456.1), were amplified from COH1 genomic DNA and the chloramphenicol resistance encoding gene, *cat*, was amplified from COH1Δ*bspc* (Deng et al., 2019). Overlap extension PCR was performed to ligate the three fragments using Failsafe PreMix B and polymerase (Lucigen) and primers containing flanking *Sac*II and *Xho*I cut sites. Each Failsafe Premix contains 100 mM Tris–HCl (pH 8.3), 100 mM KCl, and 400 μM of each dNTP with varying concentrations of MgCl_2_ and FailSafe PCR Enhancer. Deletion constructs and shuttle vector pHY304 (Jeng et al., 2003) were digested overnight with *Sac*II and *Xho*I, gel purified, ethanol precipitated, and ligated using Quick Ligase (NEB) according to the manufacturer’s protocol. The ligated reaction was transformed in MC1061 *E. coli* according to the Mclab protocol, plated on LB containing erythromycin (500 μg/mL), and allowed to grow for 3–5 days at 30°C. Transformants were confirmed using T3 and T7 primers and Q5 polymerase reagents (NEB). T3 and T7 primer sequences can be found in Supplementary Table S5. Positive transformants were maxi-prepped (Qiagen) and ∼1 mg/mL plasmid was transformed into competent GBS cells via electroporation at 1500 V and plating on THA plates containing erythromycin (5 μg/mL). The first crossover of the plasmid into the GBS chromosome was induced by diluting the plasmid-expressing strain 1:1000 into THB with 5 μg/mL erythromycin, growing overnight at 37°C, and plating on THA containing erythromycin (5 μg/mL). First crossover transformants were confirmed using primers 5flankF and MR20 or MR21 and 3flankR (Supplementary Table S5). The second crossover event was induced by diluting first crossover transformants 1:1000 in THB at 30°C. Positive second crossover transformants were identified by growth in chloramphenicol (2 μg/mL), but not erythromycin (5 μg/mL), indicating excision of the plasmid but retention of the deletion construct in the genome. Primers used can be found in Supplementary Table S5.

Complementation of *cas9* was performed by amplifying *cas9* from Topo vector PCR2.1 + *cas9* using primers 5001 and 3001, which contain SacII and BamH1 sites, respectively. The *cas9* PCR product and overexpression plasmid pDCErm were digested with SacII and BamH1 for 30 min and calf-intestinal phosphatase (Promega) was added to the pDCErm reaction for 30 min at 37°C to prevent plasmid re-ligation. These products were ligated using Quick Ligase (NEB) and transformed into MC1061. The transformation was plated on THA with erythromycin (5 μg/mL). The plasmid containing *cas9* was electroporated into *cas9* deficient strains of GBS as described above. Vector controls of the mutant strains were made by transforming empty pDCErm vector into competent mutant GBS strains as described above.

### Growth of GBS Strains in Rich Media

To assess growth of GBS strains in rich media, WT COH1 and Δ*cas9* mutant strains were grown to 1 × 10^8^ CFU/mL and frozen stocks were made at a final concentration of 16% glycerol. Frozen stocks were centrifuged, resuspended in the original volume of THB and added to 4 mL of THB at a 1:100 dilution, rendering 1 × 10^6^ CFU/mL starting cultures. Cultures were grown statically at 37°C and growth was assessed by CFU counts every hour for 7 h by serial dilution of the cultures.

### Assessment of GBS Capsule Expression by Flow Cytometry

Serotype III capsule production was assessed in COH1 background strains as previously described (Deng et al., 2019) with slight modifications. Frozen stocks of mid-log grown WT COH1 and Δ*cas9* mutant strains were centrifuged, washed in HBC buffer (1 × HBSS without magnesium or calcium, 0.5% Bovine serum albumin, 2.2 mM CaCl_2_), and normalized to approximately 5 × 10^5^ CFU/well. Bacteria were incubated for 30 min (at 4°C with shaking) with anti-serotype III IgM monoclonal antibody or with anti-serotype Ia IgM monoclonal antibody as an isotype control at final dilutions of 1:20,000 in HBC. Bacteria were centrifuged, washed in HBC twice, and incubated for 30 min (at 4°C with shaking) with donkey anti-mouse IgM-Alexa Fluor 647 secondary antibody (Jackson ImmunoResearch Laboratories Inc.) at a 1:2000 final dilution in HBC. Bacteria were centrifuged, washed twice, and resuspend in HBC for flow analysis on a FACSCalibur flow cytometer. Flow cytometric data was analyzed using FlowJo v10.

### Murine Model of GBS Vaginal Colonization

Our murine model of GBS vaginal colonization has been described previously (Patras and Doran, 2016). Briefly, female CD1 (Charles River) and C57BL/6 (Jackson laboratories) mice age 8–12 weeks were synced with beta-estradiol at day -1 and inoculated with 1 × 10^7^ bacteria in PBS on day 0. After inoculation, mice were swabbed daily and the swab samples were serially diluted and plated for CFU counts to determine bacterial persistence. Dilutions were plated on GBS CHROMagar [SB282(B)], which allows only for the growth of GBS (in pink) and *Enterococcus* spp. (in blue). These experiments were approved by the committee on the use and care of animals at the University of Colorado-Anschutz Medical Campus in our protocol #00316.

### Murine Model of GBS Hematogenous Meningitis

The *in vivo* model of murine hematogenous meningitis was performed as previously described (Banerjee et al., 2011; Kim et al., 2015). Male, 6–8-week-old CD1 mice (Charles River) were singly-challenged intravenously with 2 × 10^8^ bacteria. At 72 h post-infection tissues were harvested, homogenized, and serially diluted to determine bacterial load. Animals were sacrificed prior to 72 h if deemed moribund in accordance with IACUC and veterinary standards. These experiments were approved by the committee on the use and care of animals at the University of Colorado-Anschutz Medical Campus in our protocol #00316.

### Adherence and Invasion of GBS to Host Cells

Adherence and invasion assays were performed as previously described (Deng et al., 2019). Briefly, cell lines were seeded into 24 well plates and grown to a complete monolayer (approximately 1 × 10^5^ cells/well). GBS was grown to mid-log phase and normalized to 1 × 10^8^ CFU/mL in PBS. 1 × 10^5^ bacteria were added to one well of host cells to achieve a MOI of 1. To assess adherence of GBS to host cells, bacteria were incubated with host cells for 30 min then the cells were washed five times with PBS. Host cells were detached with 0.25% trypsin (Thermo Fisher Scientific) and permeabilized with 0.025% Triton X-100 (Sigma) in PBS, serially diluted, and plated to quantify all cell-associated bacteria. To assess bacterial invasion, GBS was incubated with host cells for 2 h, the monolayer was washed three times with PBS and then incubated with media containing antibiotics (5 ng/μL penicillin + 100 ng/μL gentamycin) for two additional hours to kill extracellular bacteria. Host cells were then trypsinized and permeabilized and lysates were serially diluted and plated to quantify invaded bacteria.

### RNA-Seq Analysis of WT GBS and Δ*cas9* Mutant, *in vitro*

Bacterial cultures were grown in triplicate to early-, mid-, and late-logarithmic phase and were lysed by beating for 3 min at max speed on a bead beater with 0.1 mm diameter zirconia/silica beads (BioSpec Products). RNA was isolated following the manufacturer’s protocol using the Direct-Zol RNA MiniPrep Plus kit (Zymo Research). Illumina cDNA libraries were constructed and sequenced at the Broad Institute of MIT and Harvard Microbial “Omics” Core using a modified version of the RNAtag-Seq protocol (Shishkin et al., 2015) as performed in Deng et al. (2018). Approximately 1 μg of total RNA was fragmented, depleted of genomic DNA, dephosphorylated, and ligated to DNA adapters carrying 5′-AN8-3′ barcodes of known sequence with a 5′ phosphate and a 3′ blocking group. Barcoded RNAs were pooled and depleted of rRNA using a RiboZero rRNA depletion kit (Epicenter). Pools of barcoded RNAs were converted to Illumina cDNA libraries via reverse transcription of the RNA using a primer designed to be specific to the constant region of the barcoded adapter with addition of an adapter to the 3′ end of the cDNA by template switching using SMARTScribe reverse transcriptase (Clontech) as described previously (Deng et al., 2018) and PCR amplification using primers whose 5′ ends target the constant regions of the 3′ or 5′ adapters and whose 3′ ends contain the full Illumina P5 or P7 sequences. cDNA libraries were sequenced on an Illumina NextSeq 500 platform to generate paired end reads.

Coding sequences from the DESeq2 and EdgeR analyses were compared, and only transcripts determined to have an adjusted *p*-value <0.05 by both methods and a mean log 2-fold change ±1 were considered significant to reduce procedural bias. Mean transcript counts were normalized between the two methods by relative abundance transformation. Transcripts were annotated using GenBank, accession NZ_HG939456.1. COGs were assigned to dysregulated genes according to designations determined by IMG/M system at the JGI (Chen et al., 2019). Volcano plots were generated using the ggplot2 package in R and Venn diagrams were generated using the using the area-proportional Venn diagram tool (BioInfoRx).

### qRT-PCR Confirmation of RNA-Sequencing

qRT-PCR was performed as previously described (Deng et al., 2018) to confirm dysregulated genes observed in the RNA-Seq results. Bacterial strains were grown in triplicate to mid-log phase and bacterial RNA was isolated as described above with an additional DNase treatment (Turbo DNase, Invitrogen) to remove contaminating genomic DNA. cDNA was generated using the Quanta cDNA synthesis kit (Quanta biosciences) and transcript abundance was determined using PerfeCTa SYBR Green reagent. Fold changes in transcript abundance were calculated using ΔΔCT, by which target gene transcript levels were normalized to those of housekeeping gene, *gyrA*. qRT-PCR data represent the average of three cultures. qRT-primers used in this study are listed in Supplementary Table S5.

### Statistical Analysis

Statistical analysis was perform using Prism version 8.0.1 (145) for macOS (GraphPad Software, La Jolla, CA, United States) as described in the figure legends.

## Data Availability

The datasets generated in this study were uploaded to Sequence Read Archive (http://www.ncbi.nlm.nih.gov/bioproject/557104).

## Author Contributions

BS and KD designed the study and wrote the manuscript. BS, LD, KP, and GS performed the experiments. PN and GS provided the strains for the study. ZB analyzed the RNA-Seq data. All authors contributed to the manuscript revision, read, and approved the submitted version.

## Conflict of Interest Statement

The authors declare that the research was conducted in the absence of any commercial or financial relationships that could be construed as a potential conflict of interest.
