# Supplementary figures and images for: Cas9 Contributes to Group B Streptococcal Colonization and Disease
Source: Front Microbiol. 2019 Aug 21;10:1930. doi: 10.3389/fmicb.2019.01930 (PMC6712506; doi:10.3389/fmicb.2019.01930)

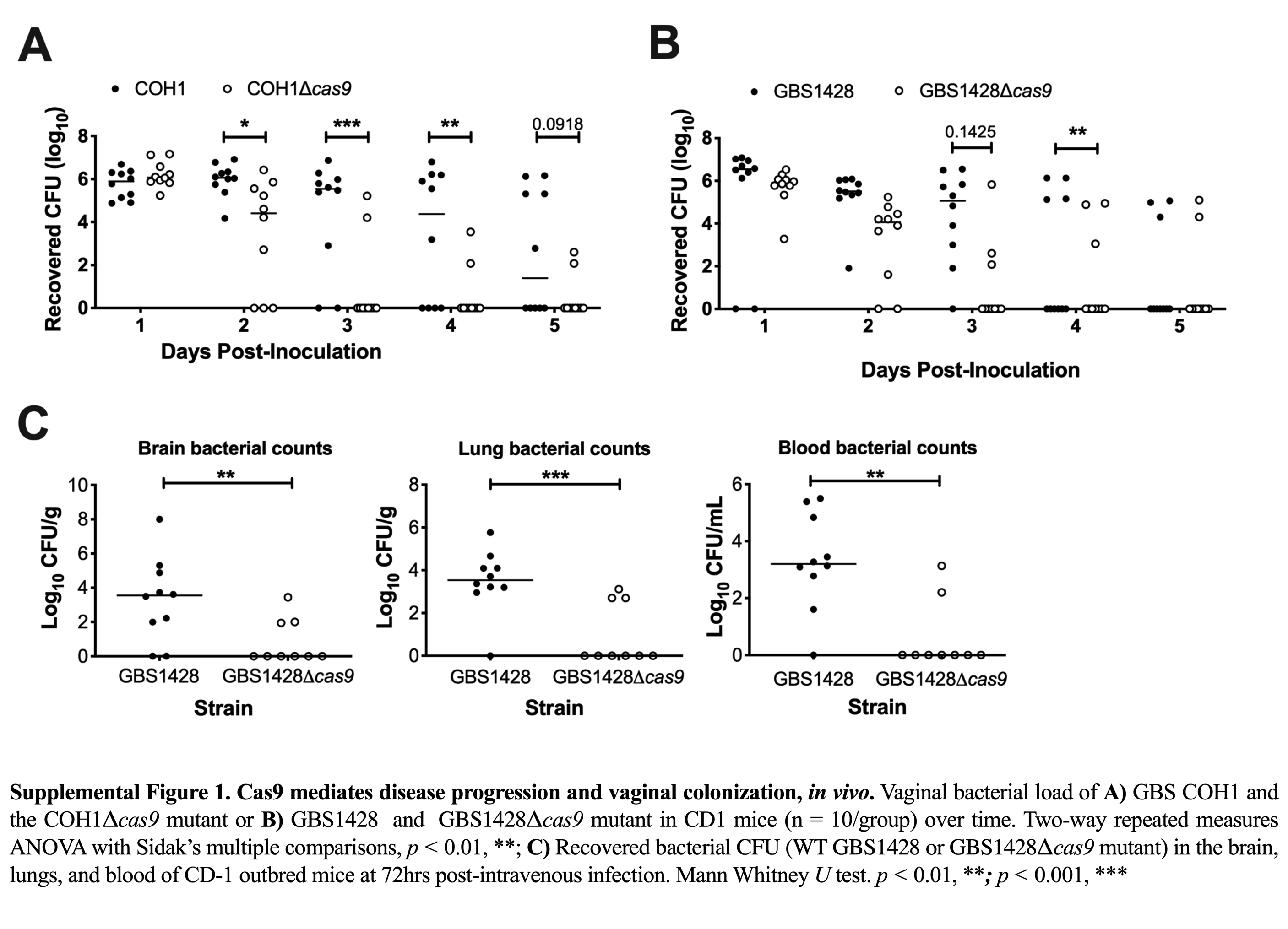

Supplement: Supplementary file 1 [file Image_1.tiff]

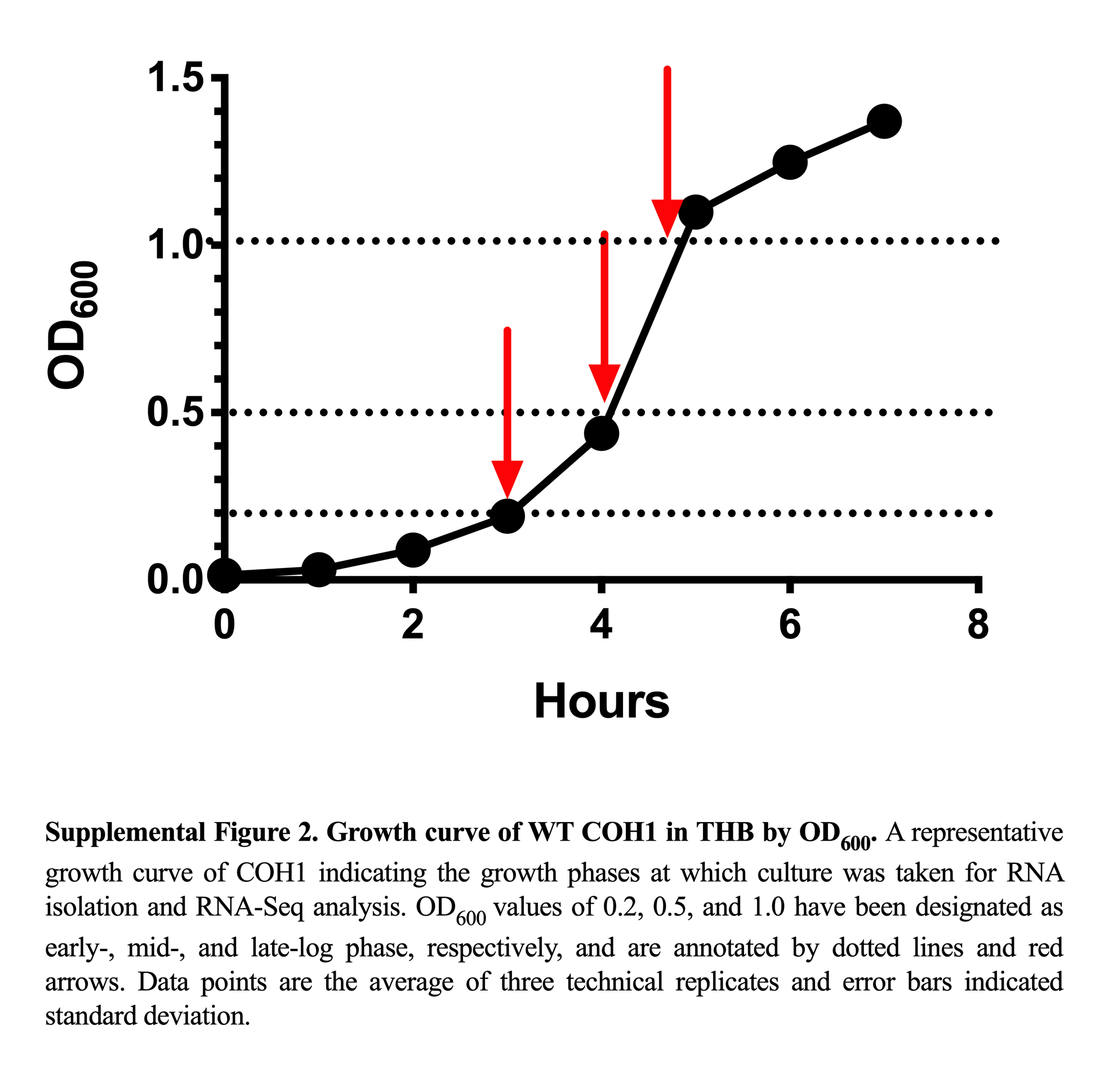

Supplement: Supplementary file 2 [file Image_2.TIFF]

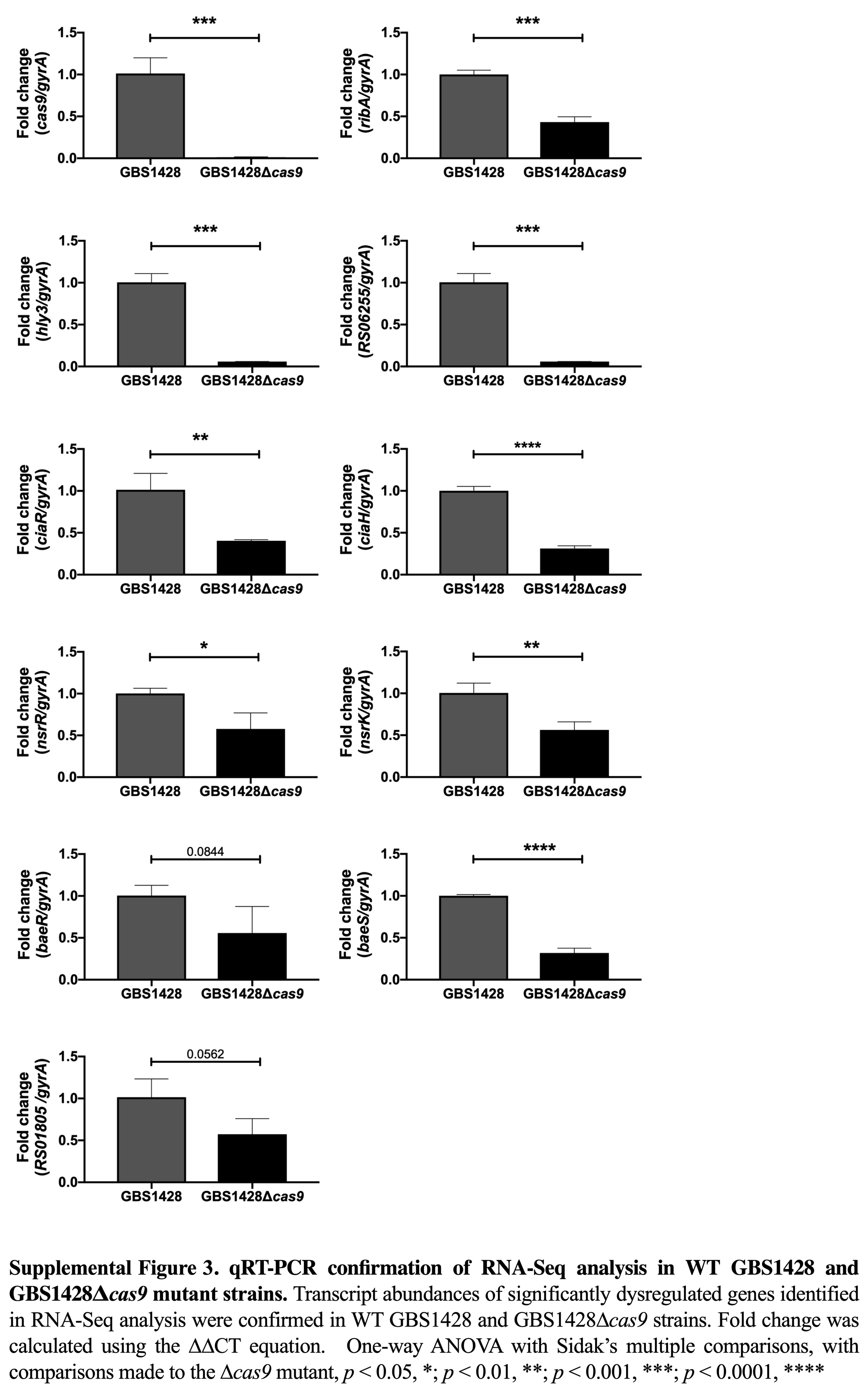

Supplement: Supplementary file 3 [file Image_3.TIFF]
